# Therapeutic drug monitoring in peritoneal dialysis: A case of nontuberculous mycobacterium catheter‐related infection treated with amikacin

**DOI:** 10.1002/ccr3.2774

**Published:** 2020-03-11

**Authors:** Sanae Kojya, Hideo Shiohira, Yoshitsugu Sunagawa, Shoji Tsuneyoshi, Kentaro Kohagura, Yusuke Ohya, Fusako Yonaha, Nobuo Hokama, Katsunori Nakamura

**Affiliations:** ^1^ Department of Pharmacy University of the Ryukyus Hospital Okinawa Japan; ^2^ Department of Cardiovascular Medicine, Nephrology and Neurology Graduate School of Medicine University of the Ryukyus Okinawa Japan; ^3^ Dialysis Unit University of the Ryukyus Hospital Okinawa Japan

**Keywords:** amikacin, nontuberculous mycobacterium, peritoneal dialysis, therapeutic drug monitoring

## Abstract

The pharmacokinetics of amikacin makes it difficult to predict the appropriate dosing to avoid harmful side effects in patients undergoing continuous ambulatory peritoneal dialysis (CAPD). The implementation of therapeutic drug monitoring may be useful in controlling amikacin serum concentrations in patients receiving CAPD.

## Graphical abstract

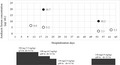

## 1 WHAT IS KNOWN AND OBJECTIVE

Continuous ambulatory peritoneal dialysis (CAPD) is an important modality for the management of end‐stage renal disease (ESRD). In Japan, it was reported that 9445 patients received CAPD therapy in 2018.^1^ PD is associated with various infectious complications, such as exit‐site infection (ESI), tunnel infection (TI), and peritonitis.^2^, ^3^ Although nontuberculous mycobacteria (NTM) are abundant in natural environments, such as soil and water, they are rarely diagnosed and documented in hospital cases.^4^, ^5^, ^6^, ^7^, ^8^ NTM (nontuberculous mycobacterium) can cause culture‐negative peritonitis, and one Japanese institution reports a frequency of 6.8%.^9^ In many cases, aminoglycoside (AG) agents such as amikacin (AMK) are used to treat NTM infections.^9^ A major side effect of AG use is renal dysfunction. Therapeutic drug monitoring (TDM) is widely recommended to control AG serum concentrations.^10^ However, for cases of NTM‐related infections such as ESI and peritonitis in CAPD patients who have end‐stage renal failure, there is little information about TDM in AG treatment. We report a case of ESI caused by NTM in a patient on CAPD who was subsequently treated with AMK which was controlled by TDM. This study was conducted in compliance with the “Ethical Guidelines for Medical and Health Research Involving Human Subjects,” and it was carried out with the approval of University of the Ryukyus Ethics Review Committee (approval number 1102).

## 2 CASE DESCRIPTION

**Figure 1:**
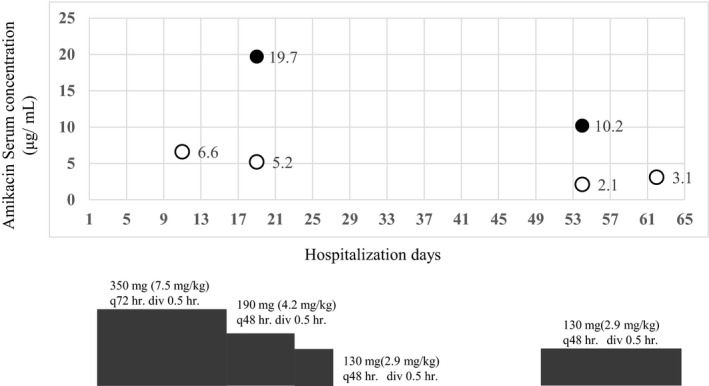
Clinical course of the patient. The result of amikacin‐therapeutic drug monitoring from intravenous (IV) administration (trough: open circles, peak: closed circles)

**Figure 2:**
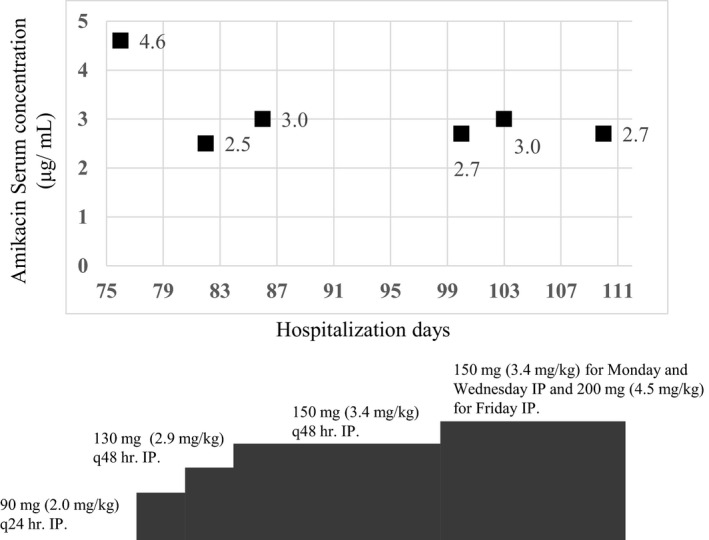
Clinical course of the patient. The result of amikacin‐therapeutic drug monitoring from intraperitoneal (IP) administration (trough before IP administration: closed squares)

A 76‐year‐old Japanese woman weighing 44.5 kg, diagnosed with IgA nephropathy, was initiated on CAPD on July 2016. Her daily PD exchange protocol consisted of 2 cycles of 1.5 L of 1.35% glucose‐based solutions (dwell time, 4 hours each). After 1 month, symptoms of exit‐site infection (dehiscence of the partial cuff, pus discharge, redness, swelling, pressure pain, and fever of 37.2°C) were observed, and she was prescribed cefaclor 750 mg/d for 7 days. However, due to a lack of clinical improvement and a negative pus culture, she was hospitalized for treatment of a suspected NTM infection and catheter replacement. Empirical treatment with clarithromycin (CAM) 500 mg/d, moxifloxacin (MFLX) 400 mg/d, imipenem‐cilastatin 250 mg, q24 hours, and AMK 350 mg q72 hours (7.5 mg/kg) intravenous administration (IV) was started on the 1st day of hospitalization. On day 8 of hospitalization, NTM infection was confirmed with a positive mycobacterium culture; however, the strain could not be identified. Since the serum concentration of AMK is known to be closely related to side effects, such as nephrotoxicity, we performed TDM. On days 11 and 19 of hospitalization, the AMK trough serum concentration was 6.6 μg/mL and 5.2 μg/mL, respectively (Figure 1), both exceeding the therapeutic range (<4 μg/mL). Amikacin dosage was adjusted to 130 mg q48 hours. The symptoms improved on day 27 of hospitalization. AMK was discontinued, but oral administration (PO) of ethambutol and levofloxacin was continued. However, AMK was resumed on day 51 of hospitalization when the patient's fever relapsed. TDM was also resumed, and the trough serum concentrations of AMK were 2.1 μg/mL and 3.1 μg/mL, respectively, on days 54 and 62 of hospitalization. On day 75 of hospitalization, the AMK administration route was changed from IV to intraperitoneal administration (IP), aiming at home discharge. Amikacin dosage was started from 90 mg q24 hours IP and adjusted to 150 mg, for Monday and Wednesday, and 200 mg for Friday IP, following the TDM results on day 76 (4.6 μg/mL), 82 (2.5 μg/mL), and 86 (3.0 μg/mL). After a fixed dosage, AMK serum concentrations were controlled within ≦3 μg/mL (Figure 2). On the first day of hospitalization, the patient's serum creatinine was 4.62 mg/dL with a urine output of 150 mL/d, and 2 days prior to discharge, the serum creatinine was 3.68 mg/dL with a urine output of 1200 mL/d. There was no observed reduction in renal function throughout hospitalization. After discharge, the patient completed 6 months of treatment using a combination of AMK IP and CAM and MFLX PO.

## 3 DISCUSSION

Amikacin is widely known to cause renal dysfunction. In a recently published meta‐analysis on nephrotoxicity, although the authors stated that no conclusions could be drawn on the toxicity of AMK relative to other aminoglycosides, a significant risk ratio of 0.48 (95% CI 0.32, 0.72) was reported when comparing AMK with other aminoglycosides.^10^ In the literature, most AMK target ranges were for doses of 7.5 mg/kg q8‐12 hours, with target peak and trough concentrations of 15‐40 mg/L and <5 mg/L, respectively, after an IV dose. The reported measured peak average concentrations were around 28 mg/L and trough concentrations around 5 mg/L.^10^ The results of actual measurement with once‐daily dosing were 40‐45 mg/L peak and 1‐2 mg/L trough concentrations, respectively.^11^ These reports suggest that the AMK trough concentration is controlled at a lower level than the targeted concentrations in clinical practice. Cumulative AUC and dosing interval were reported to be significantly related to the probability of developing nephrotoxicity. In any given cumulative AUC, the risk of toxicity was lower for once‐daily dosing than traditional multiple dosing.^12^ The pharmacokinetics of AMK in CAPD patients in doses of 7.5 mg/kg, q72 hours was significantly different for IV and IP, with a trend of higher serum concentrations in the IV route in up to 72 hours after dosing.^13^ In our patient, the final adjusted regimen of IV doses were set lower than that of IP, and AMK concentrations were controlled at <5 μg/mL for both methods. The final adjusted dose by TDM for this patient was set at 2.9 mg/kg, q48 hours for IV. This dosage was lower than the reported 7.5 mg/ kg, q72 hours and average serum concentrations (11.5 ± 3.4 μg/mL for 72 hours after administration) as reported in the literature.^13^ Also, the IP dose for our patient was set at 500 mg/wk. We considered that the adjusted dosage could avoid the possibility of overdosing with the AMK IP dosing method recommended by the IPSD guidelines (2 mg/kg daily, 623 mg/wk).^3^

## 4 WHAT IS NEW AND CONCLUSION

This case report strongly suggests that it is difficult to control AMK serum concentrations in both the IV and IP routes when using a uniform administration method for patients undergoing CAPD.

## CONFLICT OF INTEREST

The authors declare that there are no conflicts of interest.

## AUTHOR CONTRIBUTIONS

KS, HS and NK: wrote the manuscript; KS, HS, SY, TS, KG and OY: participated in patient's evaluation and treatment; YF and HN: supervised the writing of the manuscript; all authors read and approved the final manuscript.

## References

[1] 2018 Annual Dialysis Data Report, JSDT Renal Data Registry. Journal of Japanese Society for Dialysis Therapy. 2019; 52: 679‐754. (Written in Japanese).

[2] Li PK , Szeto CC , Piraino B , et al. ISPD peritonitis recommendations: 2016 update on prevention and treatment. Perit Dial Int. 2016;36:481‐508. 27282851 10.3747/pdi.2016.00078 PMC5033625

[3] Szeto CC , Li PK , Johnson DW , et al. ISPD catheter‐related infection recommendations: 2017 update. Perit Dial Int. 2017;37:141‐154. 28360365 10.3747/pdi.2016.00120

[4] Yoshitaka M , Tomomi U , Akiko Y , Akiko T , Naoto I , Tatsuo S . Nontuberculous mycobacterial peritonitis in a patient undergoing continuous ambulatory peritoneal dialysis. J Rural Med. 2009;4:75‐79.

[5] Patil R , Patil T , Schenfeld L , Massoud S . Mycobacterium porcinum peritonitis in a patient on continuous ambulatory peritoneal dialysis. J Gen Intern Med. 2011;26:346‐348. 21104454 10.1007/s11606-010-1571-y PMC3043184

[6] Yang TK , Lee JJ , Lu PL , Kuo HT , Kuo MC , Chen HC . Peritoneal dialysis‐associated peritonitis caused by mycobacterium abscessus. Perit Dial Int. 2015;35:369‐371. 26015424 10.3747/pdi.2014.00012 PMC4444000

[7] Fujikura H , Kasahara K , Ogawa Y , et al. Mycobacterium wolinskyi peritonitis after peritoneal catheter embedment surgery. Intern Med. 2017;56:3097‐3101. 28943571 10.2169/internalmedicine.8871-17 PMC5725868

[8] Yoshimura R , Kawanishi M , Fujii S , et al. Peritoneal dialysis‐associated infection caused by Mycobacterium abscessus: a case report. BMC Nephrol. 2018;19:341. 30497395 10.1186/s12882-018-1148-2 PMC6267060

[9] Washida N , Itoh H . The role of non‐tuberculous mycobacteria in peritoneal dialysis‐related infections: a literature review. Contrib Nephrol. 2018;196:155‐161. 30041221 10.1159/000485716

[10] Jenkins A , Thomson AH , Brown NM , et al. Amikacin use and therapeutic drug monitoring in adults: do dose regimens and drug exposures affect either outcome or adverse events? A systematic review. J Antimicrob Chemother. 2016;71:2754‐2759. 27494904 10.1093/jac/dkw250

[11] Maller R , Ahrne H , Holmen C , Lausen I , Nilsson LE , Smedjegård J . Once‐ versus twice‐daily amikacin regimen: efficacy and safety in systemic gram‐negative infections. Scandinavian Amikacin Once Daily Study Group. J Antimicrob Chemother. 1993;31:939‐948. 8360131 10.1093/jac/31.6.939

[12] Murry KR , McKinnon PS , Mitrzyk B , Rybak MJ . Pharmacodynamic characterization of nephrotoxicity associated with once‐daily aminoglycoside. Pharmacotherapy. 1999;19:1252‐1260. 10555931 10.1592/phco.19.16.1252.30876

[13] Smeltzer BD , Schwartzman MS , Bertino Jr JS . Amikacin pharmacokinetics during continuous ambulatory peritoneal dialysis. Antimicrob Agents Chemother. 1988;32:236‐240. 3364945 10.1128/aac.32.2.236 PMC172141

